# Lupus-associated vasculitis manifesting as acute appendicitis in a 16 year old girl

**DOI:** 10.1186/1546-0096-6-10

**Published:** 2008-06-27

**Authors:** Christina Cellini, Syed A Hoda, Nitsana Spigland

**Affiliations:** 1 Department of Surgery, New York Presbyterian Hospital-Weill Cornell Medical College, New York, USA; 2 Department of Pathology, New York Presbyterian Hospital-Weill Cornell Medical College, New York, USA

## Abstract

A 16 year old female with systemic lupus erythematosus presents with acute appendicitis. Final pathologic analysis of the appendix describes a lupus-associated vasculitis.

## Introduction

Acute abdomen in patients with systemic lupus erythematosus (SLE) can pose a diagnostic and therapeutic challenge. Most of these patients are on chronic steroid treatment which may mask symptoms and lead to delay in diagnosis. Delay in diagnosis and treatment can increase mortality. Lupus-associated vasculitis is reported to be the cause of acute abdomen in up to 60% of SLE patients [1]. We report a case of a female patient who was referred to us with symptoms of acute abdomen due to appendicitis associated with vasculitis.

## Case Report

**Figure 1:**
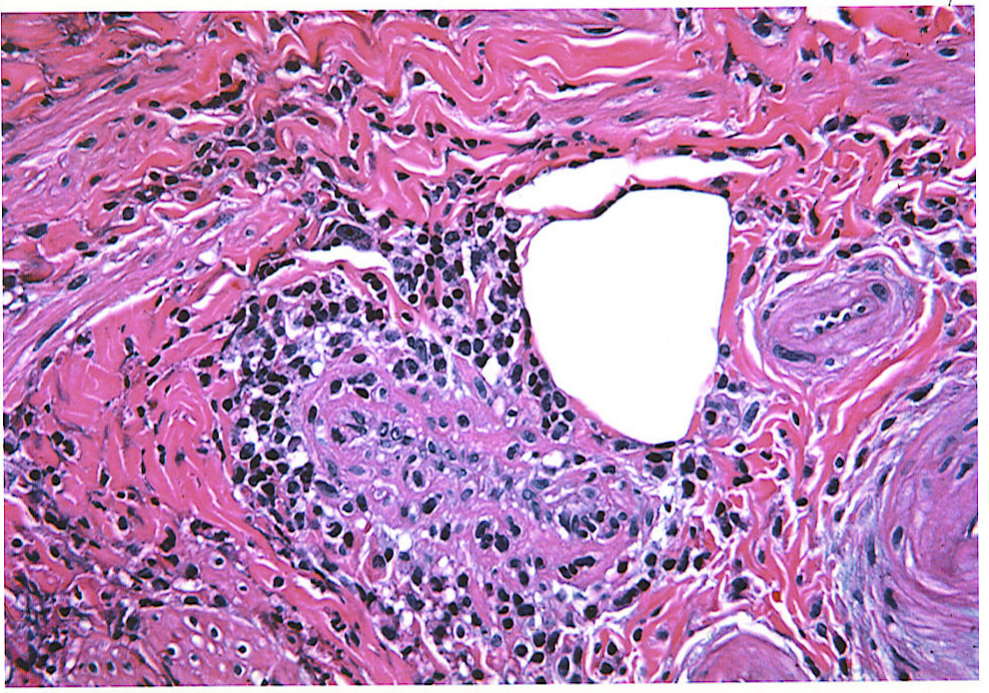
Acute and chronic appendicitis showing a small artery with transmural infiltration of lymphocytes (arrow) consistent with small vessel vasculitis.

**Figure 2:**
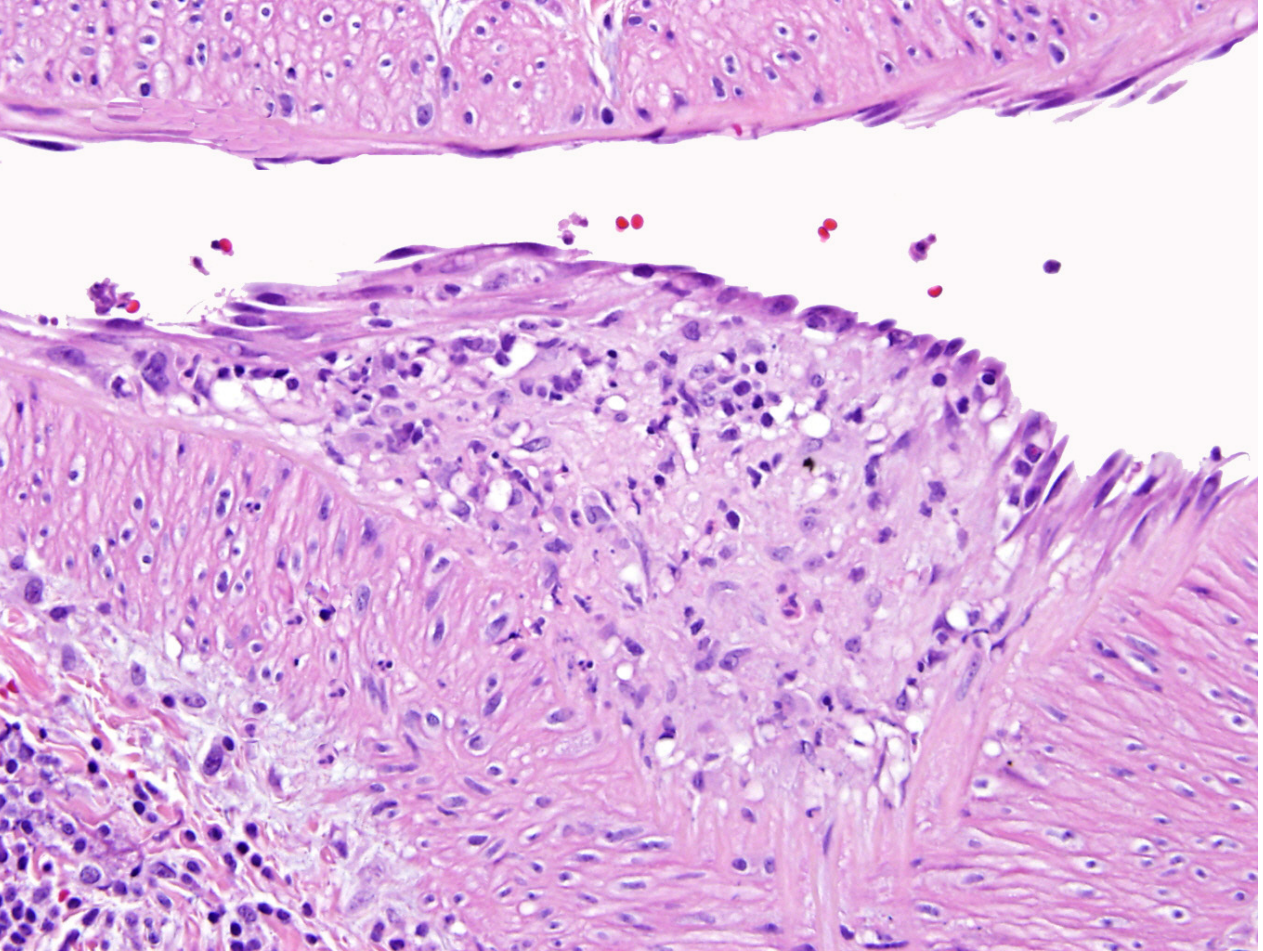
**Large vessel vasculitis evident by active endothelialitis (Fig 2), and by neutrophilic and lymphocytic infiltration of the wall (Fig. 3)**. Note fat necrosis in the vicinity of the large vessel vasculitis.

**Figure 3:**
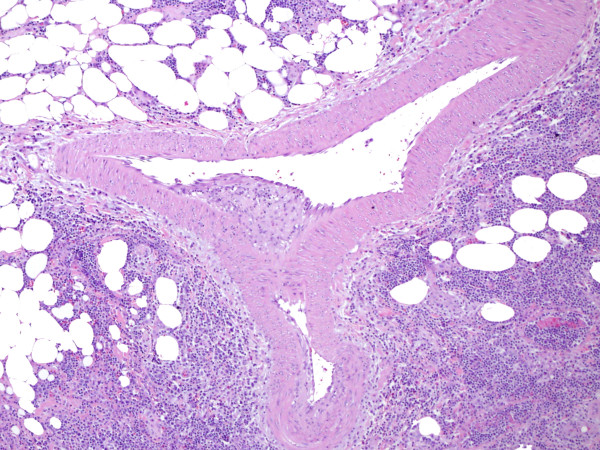
**Large vessel vasculitis evident by active endothelialitis (Fig 2), and by neutrophilic and lymphocytic infiltration of the wall (Fig. 3)**. Note fat necrosis in the vicinity of the large vessel vasculitis.

**Figure 4:**
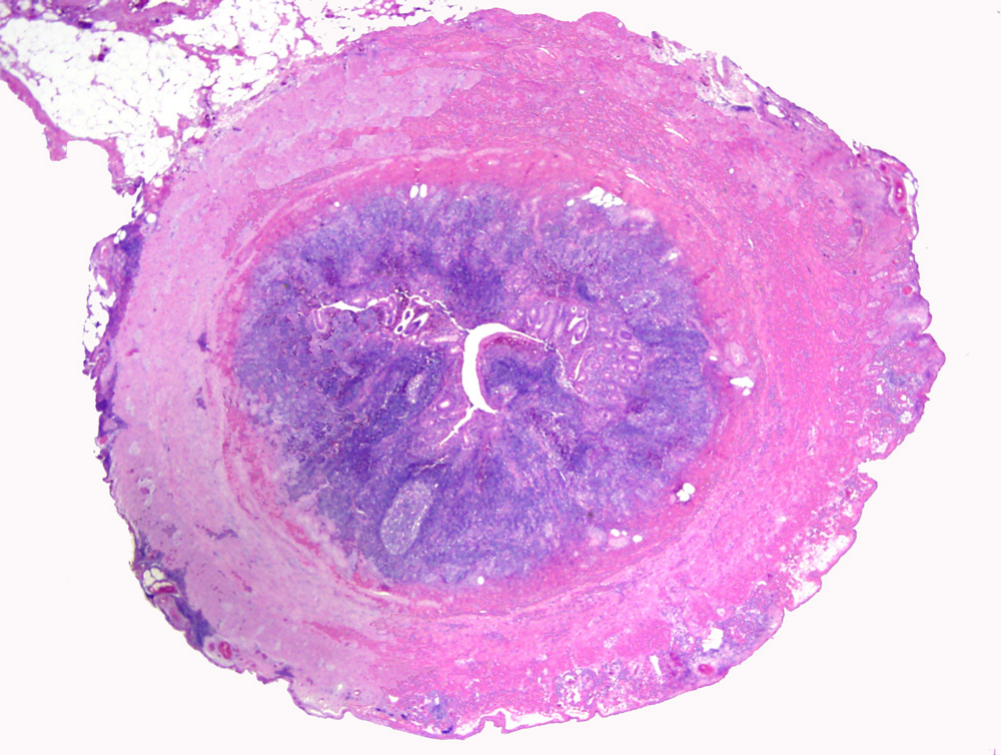
Most sections of the vermiform appendix *per se *showed no histological abnormality, and there was no significant intramural inflammation.

R.S. is a 16 year old female with a past medical history significant for systemic lupus erythematosus who presented to the ER with a one day history of right lower quadrant pain. The pain was described as constant and sharp, becoming steadily worse over the course of a day. She reported a decrease in appetite since the onset of symptoms. Past medical history was significant for SLE with biopsy- proven lupus glomerulonephritis, hypertension and hypothyroidism for which she was taking Prednisone 15 mg each day, Vasotec and Synthroid, respectively. On exam the patient was afebrile with stable vital signs. She did not appear to be in any distress. Her abdomen was obese, and non-distended. She was moderately tender to palpation in the right lower quadrant but did not exhibit any signs of rebound or guarding. Her laboratory values were significant for a WBC count of 10.2 with 84% neutrophils and a urinalysis that was positive for 5–10 WBC/hpf and moderate amount of bacteria. A CT scan of the abdomen and pelvis was obtained which revealed a slightly prominent, hyperemic, non-air or fluid filled appendix with trace pelvic ascites. The patient was then brought to the OR and underwent an open appendectomy. Intra-operatively, the appendix was inflamed with a walled off perforation into the mesentery. The appendix was removed and sent to pathology. The appendix measured 10 cm in length and 1.0 cm in greatest diameter. Upon gross examination and serial sectioning, the serosal aspect of the appendix was focally edematous and congested. The wall of the appendix was grossly unremarkable, and its lumen was empty. The entire appendix was submitted for histological evaluation and showed small vessel vasculitis and large vessel vasculitis. Small vessel vasculitis was characterized by transmural infiltration of lymphocytes (Fig 1). Large vessel vasculitis was evident by active endothelialitis (Fig 2), and by neutrophilic and lymphocytic infiltration of the wall (Fig 3). Focally, the perivascular tissue was inflamed and had undergone fat necrosis. The native appendix was essentially unremarkable, and showed no significant intramural inflammation (Fig. 4). The histological appearance was more characteristic of lupus vasculitis involving periappendiceal tissue rather than *de novo *acute appendicitis.

## Discussion

Patients with SLE presenting with an acute abdomen pose a unique diagnostic dilemma for surgeons, as the differential diagnosis is diverse and often difficult to make. Usually these patients are on chronic steroid treatment making physical exam and laboratory values unreliable therefore leading to a delay in diagnosis and treatment. Surgical intervention is fraught with many potential complications especially in patients with severe disease activity who require chronic steroid and immunosuppressive therapies. Analysis of lupus patients with an acute surgical abdomen failed to identify any clinical, laboratory or radiological features that could reliably aid in early diagnosis and management [2]. In addition it can be difficult to assess whether the cause of the pain is secondary to lupus peritonitis or to an acute surgical process [3].

Studies have found that vasculitis is the cause of acute abdomen in 35–60% of cases of SLE [1,4-6]. Causes of vasculitis- associated acute abdomen in SLE patients include intestinal ischemia and necrosis, pancreatitis and cholecystitis. Acute appendicitis is usually considered a non-SLE-related cause of acute abdomen and is considered separate from the vasculitits- associated cases. Our case, however, suggests that there might be a cause and effect relationship between the two. Review of the literature reveals two European case reports of two female patients in their twenties with SLE presenting with abdominal complaints which ultimately were the result of appendicitis following autoimmune vasculitis [7,8]. Few other reports exist linking autoimmune associated vasculitis with appendicitis, cholecystitis, bowel perforation or ischemia [1,9-12].

The typical proposed pathogenesis of appendicitis is obstruction causing increased intraluminal pressure and subsequent collapse of the draining vessels. The ischemic injury that follows favors bacterial proliferation and additional inflammatory exudate and edema leading to the typical clinical manifestations of appendicitis. It is theorized that the lupus-associated vasculitis can follow a similar pathogenetic pathway by causing ischemia, bacterial proliferation and inflammation [13].

Regardless of pathology, delay in diagnosis can be dangerous and a high index of suspicion must always be maintained when dealing with such patients. Early laparotomy shows an increase in survival rate [4]. Emergent diagnostic laparoscopy has been advocated by some as a useful way to diagnose and allow for treatment of acute abdomen in SLE [14].
